# The second confirmed record of the scorpion genus *Chactas* Gervais, 1844 (Scorpiones, Chactidae) from Ecuador with description of a new species from the Amazonian Province of Sucumbíos

**DOI:** 10.3897/zookeys.372.6665

**Published:** 2014-01-22

**Authors:** Wilson R. Lourenço

**Affiliations:** 1 Muséum national d’Histoire naturelle, Département Systématique et Evolution, UMR7205, CP 053, 57 rue Cuvier, 75005 Paris, France

**Keywords:** Scorpiones, *Chactas*, new species, Ecuador, Amazon region, Province of Sucumbíos

## Abstract

A new species, *Chactas moreti* **sp. n.**, is described from Ecuadorian Amazonia. It can be distinguished from *Chactas mahnerti* Lourenço, 1995, the other known species of this genus from Ecuador by its smaller body size, distinct morphometric values, overall darker blackish-brown coloration, totally smooth vesicle, and a concave anterior margin of carapace. This is the second confirmed record of the genus *Chactas* Gervais from Ecuador. The geographical pattern of distribution of the genus is also discussed.

## Introduction

The genus *Chactas* was created by Gervais (1844) for the species *Chactas vanbenedenii*, Gervais 1844 to which he indicated Colombia as the original locality. The current morphological diagnosis of species within the genus *Chactas* can be considered as rather difficult, mainly because several species are extremely similar. This most certainly led subsequent authors to indicate the presence of *Chactas vanbenedenii* in many localities of Colombia and Venezuela (Mello-Leitão 1945; González-Sponga 1978, 1984, 1996) Most of these records, however, have been based on misidentified specimens.

Even for the species described after *Chactas vanbenedenii*, the indication of precise localities was not common, and in some cases not even the original country was documented, (cf. *Chactas chrysopus* Pocock, 1893). Moreover, the original type materials are not always in a good state of preservation, further complicating a precise diagnosis of all species.

At present, the taxonomic classification of the genus *Chactas* is currently unresolved. This lack of clarification started a revision done by Kraepelin (1912), but was largely amplified by Mello-Leitão (1945) and González-Sponga (1978). Kraepelin (1912) originally suggested the existence of three natural groups of species within *Chactas*. He also defined for the species *Chactas lepturus* Thorell, four ‘varieties’, *typicus*, *keyserlingii* (species originally described by Pocock) and two new taxa, *intermedius* and *major*. Mello-Leitão (1945), in his monograph about South American scorpions, raised the ‘species groups’ to the rank of subgenera and the varieties to the rank of subspecies. González-Sponga (1978) proposed the creation of two new subgenera: *Caribeochactas* and *Andinochactas*. The creation of these new subgenera was primarily based on the relative number of trichobothria.

In my previous analysis of the genus *Chactas* (Lourenço, 1997), I suggested that the single variation on the number of trichobothria should not be considered as a consistent character for the division of a genus into subgenera. Several other scorpion genera also exhibit significant variation in the number of trichobothria (e. g. *Liocheles*, *Scorpiops*, *Euscorpiops* etc.), which did not lead to divisions in subgenera. This suggestion was not, however, retained in the Catalog of the Scorpions of the World (Sissom 2000).

As for the varieties or subspecies suggested for the species *Chactas lepturus*, the question remains totally unresolved. A precise and detailed ecological study could led to a correct definition of their status as a polytypical or polymorphic species (Lourenço 1997).

The pattern of distribution of the genus *Chactas*, seems to suggest a centre of dispersion in Colombia (Lourenço 1997), and most of the known species have been described from this country. Exceptions, in continental South America, are known from Venezuela (Gonzáles-Sponga 1996) and isolated cases have been reported from Brazil and Peru (Lourenço et al. 2005; Lourenço and Dastych 2001). Species are also known from Costa Rica, Panama and the Island of Trinidad (Sissom 2000).

The first species described from Ecuador, *Chactas camposi* Mello-Leitão, 1939 was reported without any indication of a locality (Mello-Leitão 1939). Subsequently, the same author (Mello-Leitão 1945) suggested Guayaquil as type locality. For a long time, the status of this species remained enigmatic (Lourenço 1997), but was later clarified by Ochoa and Pinto da Rocha (2012). These authors finally located the type specimen in the Museu Nacional at Rio de Janeiro and clearly demonstrated that the species actually belongs to the genus *Teuthraustes* Simon, 1878. Consequently, only one species *Chactas mahnerti* Lourenço, 1995 can be confirmed as present in Ecuador (Lourenço 1995). In this note, a second species of *Chactas* is described from the Amazonian Province of Sucumbíos.

## Methods

Illustrations and measurements were made with the aid of a Wild M5 stereo-microscope equipped with a drawing tube (camera lucida) and an ocular micrometer. Measurements follow Stahnke (1970) and are given in mm. Trichobothrial notations follow Vachon (1974) and morphological terminology mostly follows Hjelle (1990).

## Taxonomic treatment

### Family Chactidae Pocock, 1893
Genus *Chactas* Gervais, 1844

#### Chactas moreti sp. n.

http://zoobank.org/8FDFBB1B-E14C-4A41-BBAA-A3EE95BEA6C7

http://species-id.net/wiki/Chactas_moreti

Figs 1 –5 , 7–9 , 11–12 , 14–17

##### Material examined.

Ecuador, Province of Sucumbíus, San Pablo de Kantesiya, near to the Aguarico River, 20/V/1985 (J.-M. Touzet & P. Moret). Rainforest, under rotten log.

Male holotype, one male and two female paratypes. Deposited in the Muséum national d’Histoire naturelle, Paris.

##### Etymology.

Specific name honours Dr. Pierre Moret Université de Toulouse – Le Mirail, who sends us the specimens and donated it to our collections.

##### Diagnosis.

Moderate in size with the male holotype being 43.1 mm in total length and 40.6 mm for the female paratype. Coloration blackish-brown, except for the venter which is reddish-yellow and legs which are reddish. Body and appendages very weakly granulated or smooth, with minute punctation. Pectines with 8 teeth in males and 8-9 in females. Trichobothrial pattern type C neobothriotaxic ‘majorante’. Chela with 4 ventral trichobothria; patella with 5 ventral and 17 external trichobothria. Sexual dimorphism strongly marked by distinct morphometric values (see measurements after description).

Relationships. The new species can be distinguished from others in the genus *Chactas*, and in particular from *Chactas mahnerti* Lourenço, 1995 which is distributed in Ecuadorian Andes, by the following features: (i) a generally darker coloration, blackish-brown, whereas *Chactas mahnerti* is overall reddish-yellow to reddish-brown, (ii) a smaller global size and quite distinct morphometric values – see measurements after the description, (iii) metasomal segments are very weakly granulated and vesicle totally smooth in the new species, (iv) anterior margin of male carapace is concave in the new species, whereas the male carapace of *Chactas mahnerti* is convex.

Description based on holotype and paratypes.

Coloration. Generally blackish-brown. Prosoma: carapace blackish-brown. Tergites blackish-brown, paler than the carapace and with a central longitudinal yellowish stripe. Metasomal segments blackish-brown, with blackish zones over carinae; vesicle reddish-brown. Chelicerae reddish-yellow to reddish-brown with diffused variegated brownish spots; fingers uniformly dense and blackish; some teeth are reddish. Pedipalps blackish-brown; femur blackish; patella blackish-brown, darker than chela; chela hand very dark reddish-brown; finger blackish. Legs reddish-yellow. Venter and sternites reddish with some yellowish zones; pectines and genital operculum yellow to reddish-yellow.

Morphology. Anterior margin of carapace with a weak to moderate concavity; lustrous and acarinate, with minute punctation behind median eyes; furrows shallow. Sternum pentagonal, wider than long. Tergites acarinate, smooth and shiny with punctations. Pectinal tooth count 8-8 (male holotype) 8-8 (male paratype), 8-8 and 9-9 (female paratypes), fulcra absent. Sternites smooth and shiny with punctations, VII acarinate; spiracles moderate in size and oval to round in shape. Metasomal segments with a lustrous tegument; dorsal carinae weak to moderate on all segments, but better marked in males; latero-dorsal carinae vestigial on segments I to III, absent on IV; other carinae absent; segment V with small spinoid granulations on distal half of ventral aspect; vesicle smooth and lustrous. Pedipalps: Femur with dorsal internal, dorsal external and ventral internal carinae moderately to strongly marked; ventral external carina absent; dorsal and ventral faces without granulations, smooth; internal face weakly granular. Patella smooth and lustrous; dorsal internal and ventral internal carinae moderate to weak; ventral external carinae weak to vestigial; other carinae absent. Chela lustrous; ventral median and dorsal internal carinae weak; other carinae vestigial or absent; internal face with a few weak granules, other faces smooth. Dentate margins on movable and fixed fingers with a median denticle row composed of 7-8 groups of granules. Chelicerae with the dentition typical of the family Chactidae (Vachon, 1963), and with intense setation ventrally. Trichobothriotaxy type C; neobothriotaxic ‘majorante’ (Vachon 1974); chela with 4 ventral trichobothria; patella with 5 ventral and 17 external trichobothria. Ventral surface of tarsus in legs III and IV with a median series of small spines and 5-6 external and internal setae.

Morphometric values (in mm) of male holotype and female paratype. Total length (including telson) 43.1/40.6. Carapace: length 5.6/6.0; anterior width 3.8/4.2; posterior width 5.8/6.4. Mesosoma length 12.2/12.4. Metasomal segment I: length 2.5/2.3, width 2.8/3.0; II: length 2.8/2.5, width 2.5/2.5; III: length 3.3/2.6, width 2.4/2.4; IV: length 4.2/3.3, width 2.3/2.3; V: length 6.1/5.6, width 2.2/2.3, depth 1.9/1.9. Telson length 6.4/5.9. Vesicle: width 2.4/2.3, depth 2.0/1.8. Pedipalp length 25.2/21/4: femur length 6.8/5.3, width 1.8/1.9; patella length 6.8/5.3, width 1.9/2.2; chela length 11.6/10.8, width 2.9/3.4, depth 2.7/3.0; movable finger length 5.0/5.4.

Comparative morphometric values (in mm) of male holotype and female non-type of *Chactas mahnerti*. Total length (including telson) 51.9/45.4. Carapace: length 7.6/6.8; anterior width 5.0/4.4; posterior width 7.3/7.1. Mesosoma length 12.6/14.7. Metasomal segment I: length 3.3/2.5, width 4.0/2.9; II: length 3.4/2.7, width 3.4/2.7; III: length 4.0/3.1, width 3.4/2.6; IV: length 5.0/3.4, width 3.2/2.5; V: length 7.7/5.9, width 3.1/2.4, depth 2.8/2.1. Telson length 8.3/6.3. Vesicle: width 3.2/2.4, depth 2.8/2.2. Pedipalp length 35.8/24/1: femur length 9.8/6.1, width 2.3/2.3; patella length 10.1/6.2, width 2.3/2.6; chela length 15.9/11.8, width 3.2/3.6, depth 2.8/3.6; movable finger length 6.2/6.0.

**Figures 1–4.**
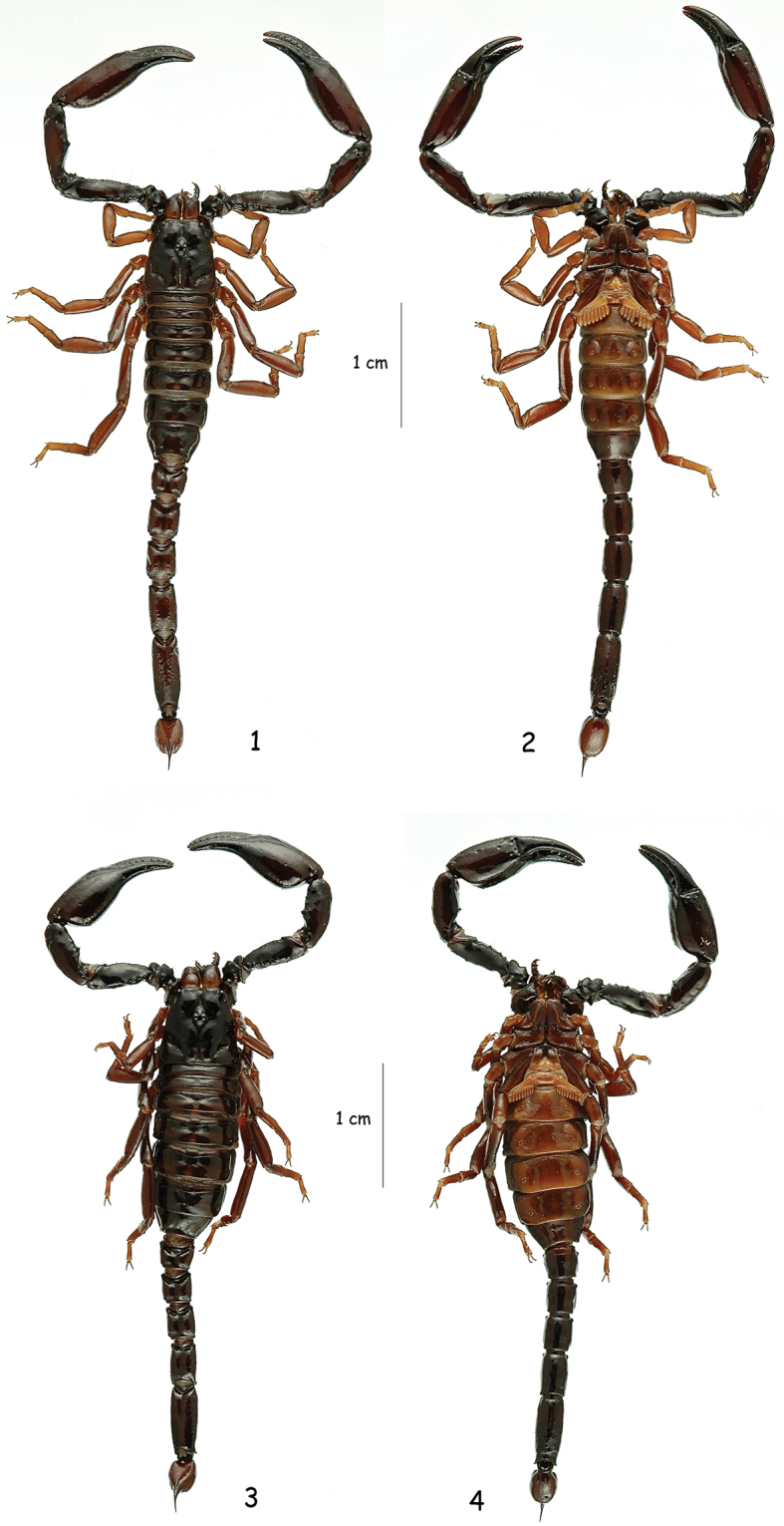
*Chactas moreti* sp. n., male holotype and female paratype. Dorsal and ventral aspects.

**Figures 5–6.**
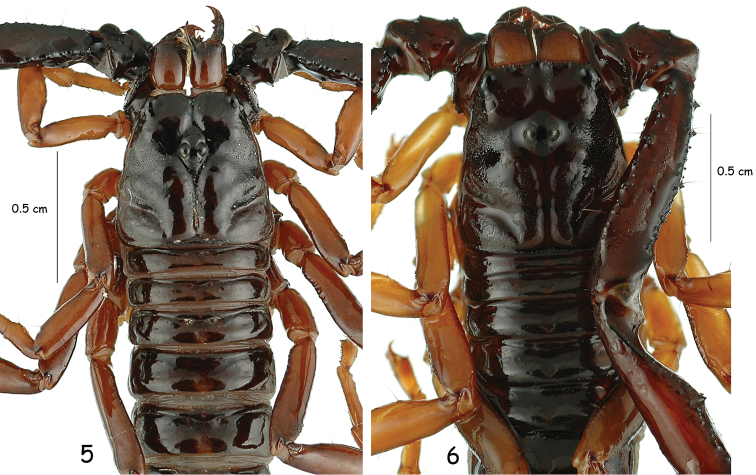
Carapace and chelicerae of male holotypes of *Chactas moreti* sp. n. and *Chactas mahnerti*.

**Figures 7–9.**
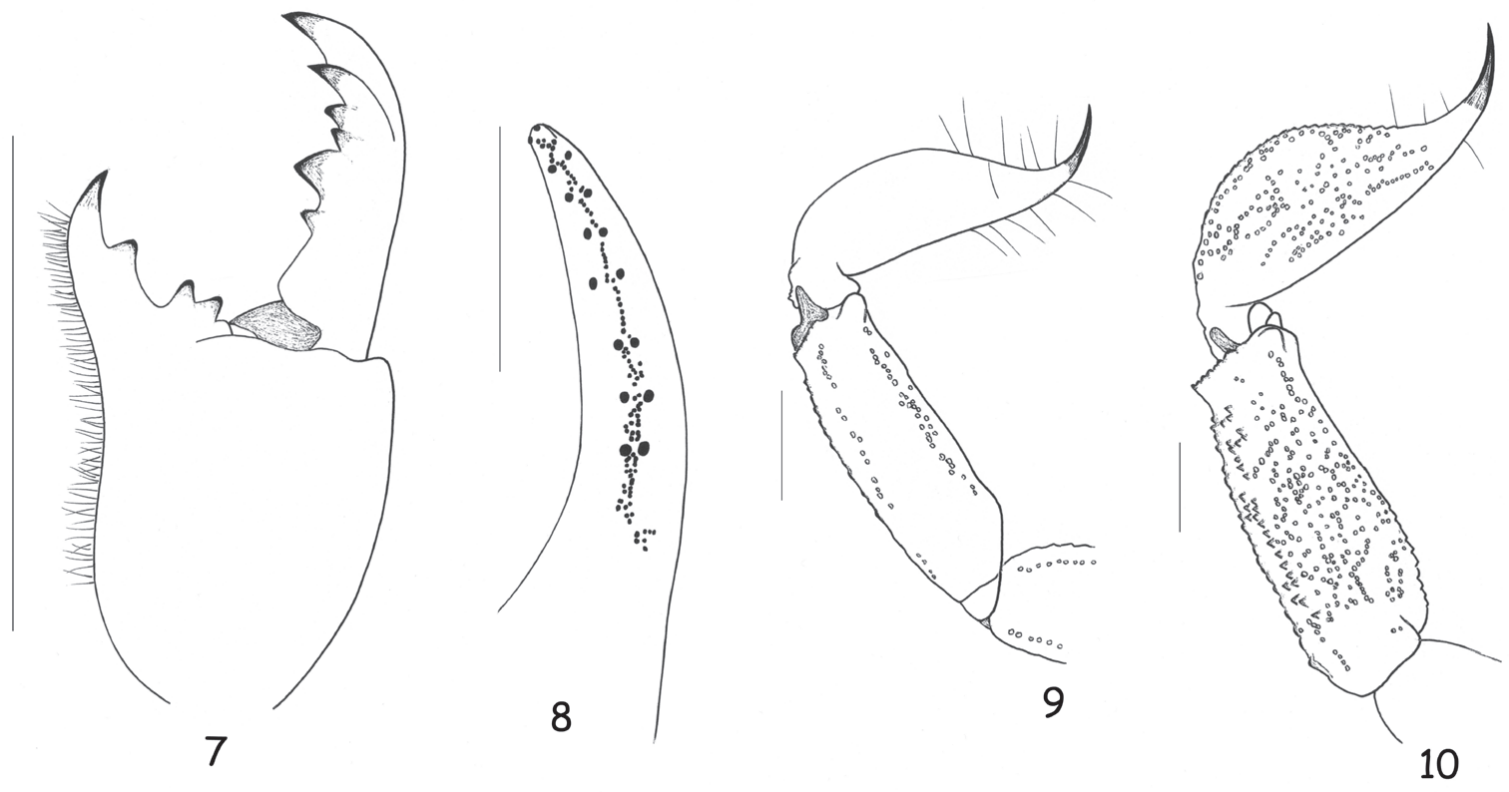
*Chactas moreti* sp. n., male holotype. **7** Chelicera, dorsal aspect **8** Disposition of granulation over the dentate margins of the pedipalp-chela movable finger **9** Metasomal segments V and telson, lateral aspect **10** Idem for *Chactas mahnerti*, male holotype (scale bars = 2 mm).

**Figures 11–17.**
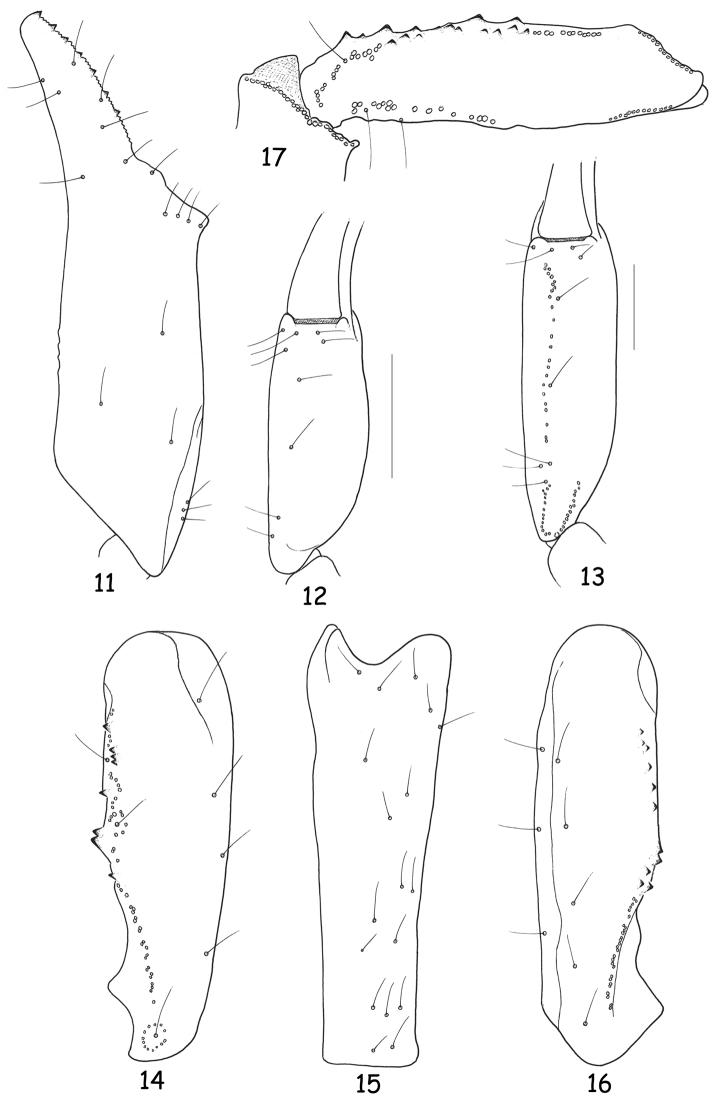
Trichobothrial pattern. **11–12, 14–17** *Chactas moreti* sp. n., male holotype **11–12** Chela, dorso-external and ventral aspects **13** Idem for *Chactas mahnerti*, male holotype **14–16** Patella, dorsal, external and ventral aspects **17** Femur, dorsal aspect (scale bars = 2 mm).

#### Key to the *Chactas* species present in Ecuador and southern Colombia:

**Table:** 

1	Total length in adult males equal or superior to 50 mm; metasomal segments strongly granulated; anterior margin of carapace convex	2
–	Total length in adult males inferior to 50 mm; metasomal segments weakly granulated to smooth; anterior margin of carapace concave	*Chactas moreti* sp. n.
2	General coloration dark to almost blackish; pedipalps granulated	*Chactas vanbenedenii*
–	General coloration yellow to reddish-yellow; pedipalps smooth and shine	*Chactas mahnerti*

**Figure 18.**
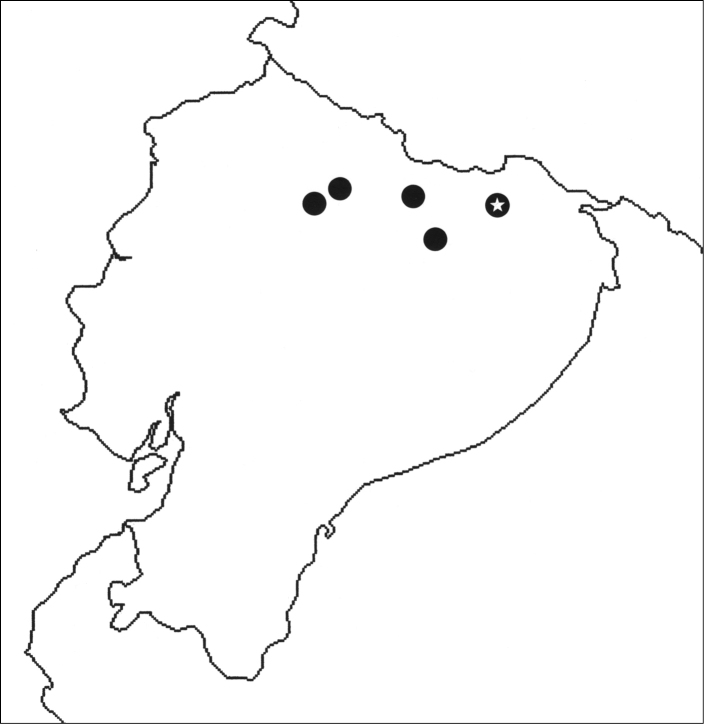
Map showing known distribution of the genus *Chactas* in Ecuador. *Chactas mahnerti* (black circles); *Chactas moreti* sp. n. (black circle with white star). Localities from West to East are: San Antonio (00°00'07"S, 78°27'21"W), La Florida (00°22'00"S, 78°30'00"W), Lumbaqui (00°03'00"S, 77°19'60"W), Coca (00°27'45"S, 76°59'03"W), San Pablo de Kantesiya (00°15'00"S, 76°26'00"W).

## Supplementary Material

XML Treatment for Chactas moreti
